# Impact of maternal fecal microbiota on the early development of neonatal gut microbial community

**DOI:** 10.3389/fped.2026.1809818

**Published:** 2026-05-29

**Authors:** Jae Youn Jo, In Ae Cho, Jin-Su Jun, Ji Sook Park

**Affiliations:** 1 Department of Obstetrics and Gynaecology, Gyeongsang National University College of Medicine, Jinju, Republic of Korea; 2 Institute of Medical Science, Gyeongsang National University, Jinju, Republic of Korea; 3 Department of Pediatrics, Gyeongsang National University Hospital, Jinju, Republic of Korea

**Keywords:** gut microbiota, maternal fecal microbiota, neonatal fecal microbiota, microbial transmission, mother-neonate pair

## Abstract

**Background:**

Maternal gut microbiota may contribute to initial colonization of neonatal fecal microbiota, but how closely neonatal fecal communities in early life reflect maternal stool is unclear.

**Methods:**

We performed 16S rRNA gene sequencing on paired maternal fecal samples collected at delivery and neonatal fecal samples collected within the first week of life from 21 mothers and 25 neonates respectively. Alpha and beta diversity and pairwise taxonomic overlap were assessed.

**Results:**

Maternal stool and early neonatal fecal samples shared a similar phylum-level profile dominated by Firmicutes. However, neonatal fecal samples had significantly lower alpha diversity and clustered separately from maternal samples, indicating a distinct community structure. Within-pair overlap supported partial maternal contribution, but many taxa in early neonatal fecal samples were not detected in maternal stool, suggesting additional perinatal or postnatal sources.

**Conclusion:**

Early neonatal fecal samples represent a low-diversity pioneer community rather than a direct replica of the maternal gut microbiota, refining models of early-life microbial transmission.

## Introduction

The human gut microbiota is a dynamic ecosystem that undergoes rapid expansion and maturation beginning at birth. Maternal microbial strains originating from the vagina, skin, and gut constitute the dominant inoculum in early life, outweighing other sources. Subsequently, the composition and functional capacity of the gut microbial community change rapidly in response to breastfeeding, weaning, and later dietary transitions (1, 2). During the neonatal period, the gut microbiota is characterized by low diversity; however, with the initiation of weaning, microbial richness and complexity progressively increase (1). Concurrently, bidirectional interactions between intestinal epithelial cells and resident microbes promote maturation of metabolic functions related to substrate utilization and contribute to the biosynthesis of essential nutrients for the host (3, 4). Large-scale cohort studies have consistently described this process as a stepwise pattern of microbial maturation (5–7). Although the exact trajectory is modulated by interindividual variability, environmental exposures, and methodological factors, a shared developmental axis has been repeatedly observed across populations (5, 7, 8).

When this developmental program is disrupted or unhealthy dietary patterns persist, accumulating evidence indicates an increased risk of non-communicable diseases, such as allergies, obesity, and inflammatory bowel disease, attributable to impaired immune maturation and dysregulation of metabolic-inflammatory pathways (1, 9, 10). In preterm infants, microbial dysbiosis often precedes clinical disease. In cases of necrotizing enterocolitis, reproducible increases or depletions of specific microbial taxa have been observed before onset (11). These associations intersect with patterns of antibiotic exposure, feeding modalities, and modes of delivery. Furthermore, recent literature suggests that early microbiota-host interactions extend to neurodevelopmental pathways, indicating that the gut-brain axis may influence long-term health outcomes (12, 13). Accordingly, strategies aimed at steering infant gut microbiota colonization toward a health-promoting trajectory are of critical importance, and identification of the key determinants underlying this process represents a necessary first step. Previous studies have demonstrated that mode of delivery, exposure to intrapartum or perinatal antibiotics, breastfeeding, timing of weaning and introduction of solid foods, and maternal diet exert substantial influences on initial microbial community assembly (1, 14, 15). Notably, studies that rigorously account for the low-biomass nature of meconium indicate that “immediate perinatal” factors, particularly delivery mode and intrapartum antibiotic administration, explain a greater proportion of variance in meconium microbiota composition than putative *in utero* influences (16). However, Dos Santos et al. reported weaker associations between perinatal factors and gut microbial community structure later in infancy, suggesting that additional determinants become increasingly influential over time (17). The precise mechanisms underlying neonatal gut microbiota community assembly remain incompletely understood.

Therefore, the present study aimed to investigate the impact of maternal fecal microbiota on early neonatal gut microbiota community formation by enrolling maternal-neonatal pairs and collecting paired maternal and neonatal fecal samples within the first week of life in the same institutional setting.

## Methods

Between January and June 2024, we prospectively enrolled mother–newborn pairs delivering at Gyeongsang National University Hospital. We included the pairs when delivery occurred at late-preterm or term gestation. Maternal samples were collected aseptically before delivery, and neonatal samples were obtained within the first week after birth, following informed consent from the parent. Immediately after collection, all specimens were frozen at −80 °C and stored in the Biobank of Gyeongsang National University Hospital (GNUH), a member of the Korea Biobank Network, until analysis. For microbiota analysis, frozen fecal samples were thawed, resuspended, homogenized, subjected to bead-beating, and centrifuged at 14,000 × g for 10 min. The resulting supernatants were diluted with nuclease-free water and used as templates for polymerase chain reaction amplification. The V3–V4 hypervariable region of the bacterial 16S rRNA gene was amplified using primers 341F and 805R. Amplicons from individual samples were combined in equimolar amounts and purified using the AMPure XP purification kit (Beckman Coulter, Indianapolis, IN, USA), following the manufacturer's protocol. Purified libraries were sequenced on an Illumina MiSeq platform with the MiSeq Reagent Kit v3 (Illumina, San Diego, CA, USA) using 2 × 250 bp paired-end reads. Extraction of bacterial DNA from fecal samples for 16S rRNA gene sequencing and initial taxonomic assignment was performed by a commercial provider (CJ Bioscience, Seoul, Korea). Taxonomic profiling of bacterial communities was conducted with EzBioCloud's Microbiome Taxonomic Profiling (MTP) cloud pipeline using the PKSSU4.0 reference database. Organisms sharing ≥95% average nucleotide identity were grouped into a single “species group,” as individual members cannot be reliably distinguished at the sequence identity thresholds typically applied in 16S rRNA gene–based profiling. A comprehensive list of conventional species names corresponding to each species group is available on the EzBioCloud taxonomy page (https://www.ezbiocloud.net/mtp/taxonomy).

To enable comparison of *α*-diversity indices among samples, read counts were rarefied to 1,000 reads per sample, representing the lowest sequencing depth in the dataset. For *β*-diversity analyses, read abundances were adjusted for 16S rRNA gene copy number variation to reduce compositional bias. Species richness was estimated using an abundance-based coverage estimator (ACE) and by enumerating observed operational taxonomic units (OTUs). Overall diversity was summarized using the NPShannon index, while phylogenetic diversity was calculated from the OTU occurrence matrix. Differences in OTU composition between samples were quantified using Jensen-Shannon and visualized with principal coordinate analysis (PCoA) combined with K-means clustering.

We also obtained demographic and perinatal factors, including delivery mode, gestational age at birth, maternal weight, offspring birth weight, use of antibiotics, and feeding methods of the mother and baby pair from the medical records. Differences in fecal microbial composition were analyzed according to these factors to investigate potential associations with neonatal gut microbiota patterns. Additional subgroup heatmaps stratified by neonatal antibiotic exposure, feeding type, and delivery mode were generated using the same EzBioCloud MTP-derived species-group level taxonomic assignments as in the primary analysis.

Continuous variables are presented as means and standard deviations (SD), and categorical variables are expressed as numbers and percentages. Maternal or neonatal factors were analyzed using Fisher's exact test or the Mann–Whitney *U*-test, as appropriate. Associations between taxonomic profile variations, diversity indices, and sample categories of mothers and neonates were assessed using the Wilcoxon rank-sum test and permutational multivariate analysis of variance (PERMANOVA). Taxonomic profiling of fecal microbiota across groups and related statistical analyses were conducted using the EzBioCloud taxonomy webpage (https://www.ezbiocloud.net/mtp/viewmyMTPSetAnalyzer) (18). Correlation analyses between demographic or perinatal factors of mother–neonate pairs and concordance of the dominant phylum were performed using logistic regression analysis in SPSS 27.0 (IBM, NY, US), and graphical representations were generated with GraphPad Prism 11.0.0 (GraphPad Software, Boston, US).

This study was approved by the Institutional Review Board of GNUH (GNUH 2025-06-007).

## Results

During the study period, a total of 21 mothers and 25 neonates with over 33 weeks of gestation were included. Detailed clinical data of the pairs are presented in Table 1. Neonatal fecal samples were collected at a mean of 4.1 ± 2.3 days after birth. Four neonates presented tachypnea after birth diagnosed transient tachypnea of newborn, and 5 neonates received antibiotics during sample collection period.

**Table 1:** Clinical characteristics of mothers and neonates participating in the study (*M* = 21, *B* = 25).

Pairs of mother and Baby	Maternal factors									Neonatal factors							
	Age (year)	Wt (kg)	BMI (kg/m^2^)	Obstetric problem	Primipara	Antibiotics	Delivery	Stool		GA (week)	BW (kg)	Sex	Tachypnea	Antibiotics	Feeding mode	Stool	
								Day	Dominant phylum						BW (kg)	Day	Dominant phylum
M1-B1	40.1	79.8	28.4	Twin	Y	N	CS	0	Fir	36.9	3.07	M	N	N	FF	0	Fir
M2-B2	31.0	88.8	34.5	GDM, PPROM	N	Y	VD	0	Pro	36.1	2.90	F	N	Y	BMF	7	Fir
M3-B3	40.2	79.4	27.8	–	N	N	CS	0	Fir	38.0	3.25	F	N	N	BMF	8	Fir
M4-B4	38.6	62.7	24.6	–	N	N	CS	−3	Fir	38.3	3.32	M	N	N	BMF	4	Fir
M5-B5	35.9	60.0	24.3	PTL	N	N	CS	−1	Fir	34.1	2.07	F	N	N	FF	5	Fir
M6-B6	37.8	49.8	19.0	FGR	N	N	CS	0	Bac	37.3	1.97	F	N	N	FF	1	Pro
M7-B7	43.6	75.4	31.3	GDM, PPROM	N	Y	CS	−1	Fir	34.0	2.01	F	N	N	FF	5	Fir
M8-B8	39.8	59.9	23.7	GDM, PE	Y	N	CS	0	Fir	35.3	1.79	M	N	N	FF	4	Fir
M9-B9	34.8	77.0	28.5	–	Y	N	VD	0	Fir	39.6	3.81	M	N	N	BMF	5	Pro
M10-B10	30.9	73.4	25.8	Twin	Y	N	CS	0	Fir	37.1	2.85	M	N	N	BMF	1	Pro
-B11										37.1	2.46	F	N	Y	FF	1	Fir
M11-B12	27.4	64.0	25.0	–	Y	N	CS	0	Fir	38.7	3.43	F	N	Y	BMF	6	Fir
M12-B13	35.0	54.0	20.1	PTL	N	N	VD	0	Pro	36.0	2.47	M	N	Y	FF	4	Pro
M13-B14	32.7	64.5	27.0	GDM	Y	N	CS	0	Bac	33.4	1.69	M	N	N	FF	5	Fir
M14-B15	25.9	55.5	24.4	–	Y	N	CS	0	Fir	38.6	3.11	F	N	N	BMF	4	Fir
M15-B16	32.9	64.7	25.5	–	Y	N	CS	0	Fir	38.0	3.24	M	N	N	BMF	7	Pro
M16-B17	35.2	63.8	26.9	IUGR	N	N	CS	0	Fir	36.4	2.48	M	Y	Y	FF	4	Pro
-B18										36.4	2.19	M	Y	N	FF	4	Fir
M17-B19	36.1	131.8	47.8	GDM	N	N	CS	0	Fir	37.7	3.54	F	N	N	FF	7	Pro
M18-B20	33.6	66.0	27.8	Twin	N	N	CS	0	Fir	36.0	2.21	F	N	N	FF	0	Pro
-B21										36.0	2.18	F	N	N	FF	0	Pro
M19-B22	41.6	41.6	30.1	–	Y	N	CS	0	Fir	38.4	3.88	F	N	N	FF	7	Pro
M20-B23	30.6	70.6	27.6	GDM, Twin	Y	N	CS	1	Fir	35.3	2.06	F	Y	N	BMF	6	Fir
-B24										35.3	2.23	M	Y	N	BMF	6	Fir
M21-B25	33.6	68.1	24.9	–	Y	N	CS	0	Fir	39.1	3.63	F	N	N	BMF	6	Chl

Wt, weight; GDM, gestational DM; PPROM, prolonged rupture of membranes defined as the rupture of the amniotic membrane more than 18 h before delivery; PTL, preterm labor pain; PE, preeclampsia; IUGR, intrauterine growth restriction; CS, Cesarean section; VD, vaginal delivery; FF, formula-fed; BMF, breast milk-fed; Fir, Firmicutes; Pro, Proteobacteria; Bac, Bacteroidetes; Chl, Chlorobi; Y, yes; N, no.

**Figure 1:**
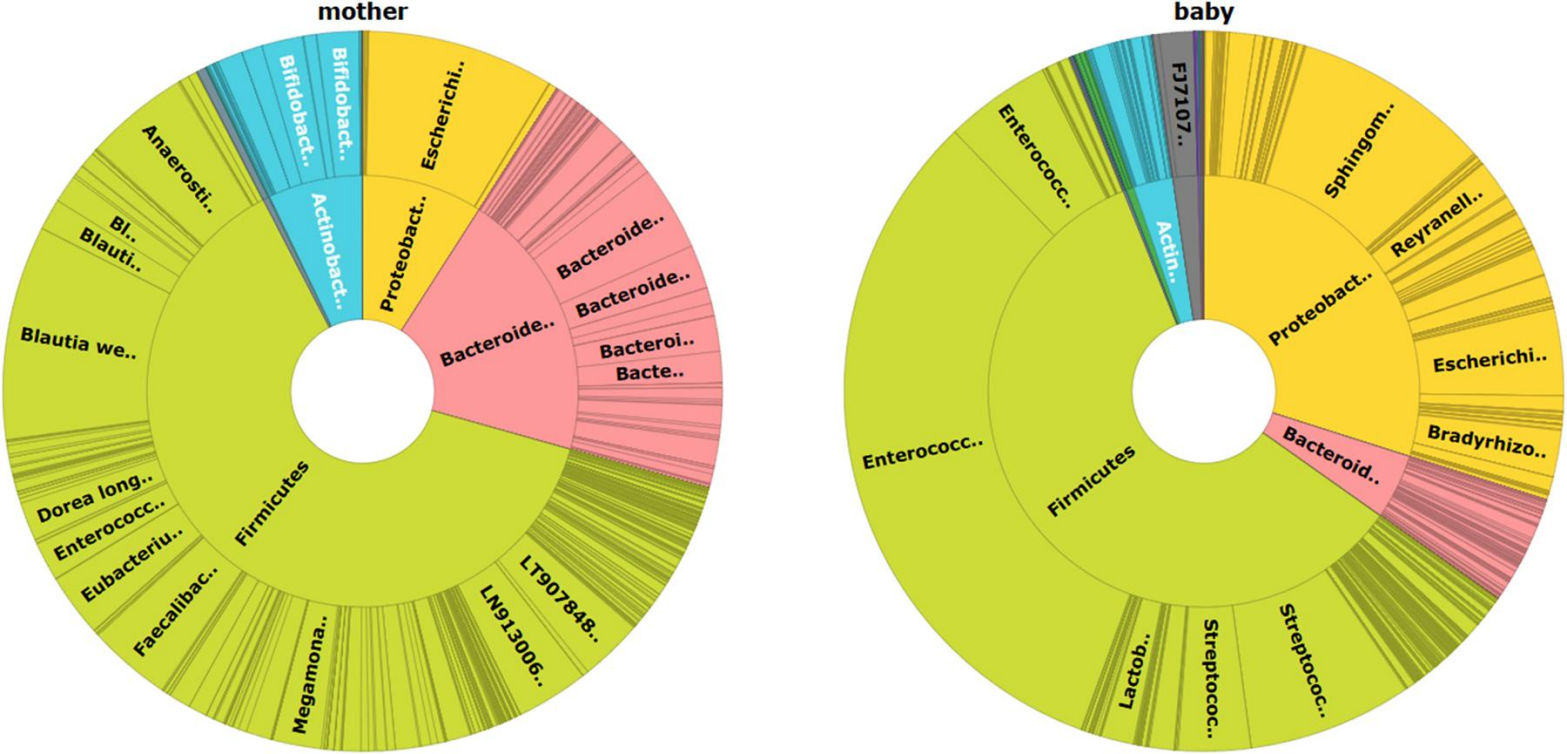
Relative abundance of the fecal microbiome in maternal stool and early neonatal fecal samples. The inner circle represents phylum-level composition, and the outer circle represents species-group level composition.

Fecal microbiota analyses were conducted for 21 mothers and 25 corresponding neonates using 16S-based MTP. *Firmicutes* was the most prevalent bacterial phylum in both mothers and neonates (Figure 1). Concordance of the dominant phylum between mothers and neonates was observed in 12 mother-neonate pairs (57.1%), including M1-B1, M3-B3, M4-B4, M5-B5, M7-B7, M8-B8, M10-B11, M11-B12, M12-B13, M14-B15, M16-B18, and M20-B23-B24. In contrast, the remaining 42.9% of pairs showed discordant dominant phyla (Table 1). Among the four mothers who delivered twins (M1, M10, M18, and M20), only one twin pair shared the same dominant phylum as their mother (M20-B23-B24), whereas the remaining twin pairs did not.

The clinical characteristics of mother-neonate pairs with concordant dominant phyla (*n* = 12) and those with discordant dominant phyla (*n* = 11) are summarized in Table 2. No statistically significant differences in maternal or neonatal factors were identified between the two groups. Maternal pre- and late-pregnancy body mass index (BMI) values were higher in the discordant dominant phylum group than those in the concordant group; however, the difference was not statistically significant. We performed logistic regression analyses to identify maternal or neonatal relating factors with phyla concordance (Table 3). Although several maternal and neonatal variables showed directional trends toward lower dominant phylum concordance, none of these associations reached statistical significance.

**Table 2:** Comparison of clinical characteristics in mother-neonate pairs between concordant and discordant dominant phylum groups.

Factors, Mean ± SD, *n* (%)		Overall	Concordant pair (*n* = 12)	Discordant pair (*n* = 11)	*P*
Maternal factors (M = 21)	Age (year)	34.0 ± 4.7	35.7 ± 6.0	34.6 ± 3.1	0.468
	Weight (kg)	71.3 ± 17.3	66.9 ± 10.2	75.7 ± 21.5	0.251
	BMI, pre-pregnancy	23.2 ± 5.4	22.3 ± 3.8	24.0 ± 6.6	0.809
	BMI at delivery	27.3 ± 5.8	25.7 ± 3.1	28.9 ± 7.3	0.197
	Primiparous	11 (52.4%)	5 (50.0)	6 (54.5)	1.000
	GDM	6 (28.6%)	3 (30.0)	3 (27.3)	1.000
	Stool collection	−0.1 ± 0.7	−0.4 ± 1.1	0.0 ± 0.0	0.159
	CS delivery	18 (85.7%)	9 (90.0)	9 (81.8)	1.000
Neonatal factors (*N* = 25)	GA (week)	36.9 ± 1.8	36.5 ± 1.6	37.1 ± 1.7	0.326
	Birth Weight (kg)	2.7 ± 0.7	2.57 ± 0.58	2.86 ± 0.76	0.347
	Male	11 (44.0%)	6 (46.2)	5 (41.7)	1.000
	Stool day	4.1 ± 2.3	4.4 ± 2.1	4.2 ± 2.9	0.794
	Breast milk-fed	11 (44.0%)	6 (46.2)	5 (41.7)	1.000
	Tachypnea	4 (16.0%)	3 (23.1)	1 (8.3)	0.593
	Antibiotics	5 (20.0%)	3 (23.1)	2 (16.7)	1.000
	Hospital duration	7.4 ± 5.2	8.0 ± 4.2	6.8 ± 6.3	0.194

*P*-values were obtained using Fisher's exact test or the Mann–Whitney test. The Overall column summarizes the full cohort (21 mothers and 25 neonates), whereas concordant and discordant columns reflect pair-based subgroup comparisons.

SD, standard deviation; M, mother; N, neonates; BMI, body mass index; GDM, gestational diabetes mellitus; CS, Cesarean section; GA, gestational age.

**Table 3:** Logistic regression analysis of maternal or neonatal clinical factors associated with concordant dominant phylum.

Factors		Exp (B)	95% CI (lower-upper)	*P*
Maternal factor	Age, year	1.038	0.853–1.264	0.708
	BMI	0.869	0.691–1.093	0.231
	Weight changes, kg	0.957	0.834–1.098	0.530
	CS delivery	0.389	0.029–5.214	0.476
	GDM	0.857	0.124–5.944	0.876
	Primi-parous	0.800	0.131–4.874	0.809
	Multi-gestation	0.500	0.037–6.683	0.600
Neonatal factor	GA, week	0.773	0.463–1.290	0.324
	Birth weight, kg	0.504	0.146–1.737	0.277
	Male	0.833	0.171–4.058	0.821
	BMF	0.833	0.171–4.058	0.821
	Tachypnea	0.303	0.027–3.407	0.334
	Postnatal antibiotics	0.667	0.091–4.889	0.690

BMI, body mass index; CS, Cesarean section; GDM, gestational diabetes mellitus; GA, gestational age; BMF, breast milk-fed.

**Figure 2:**
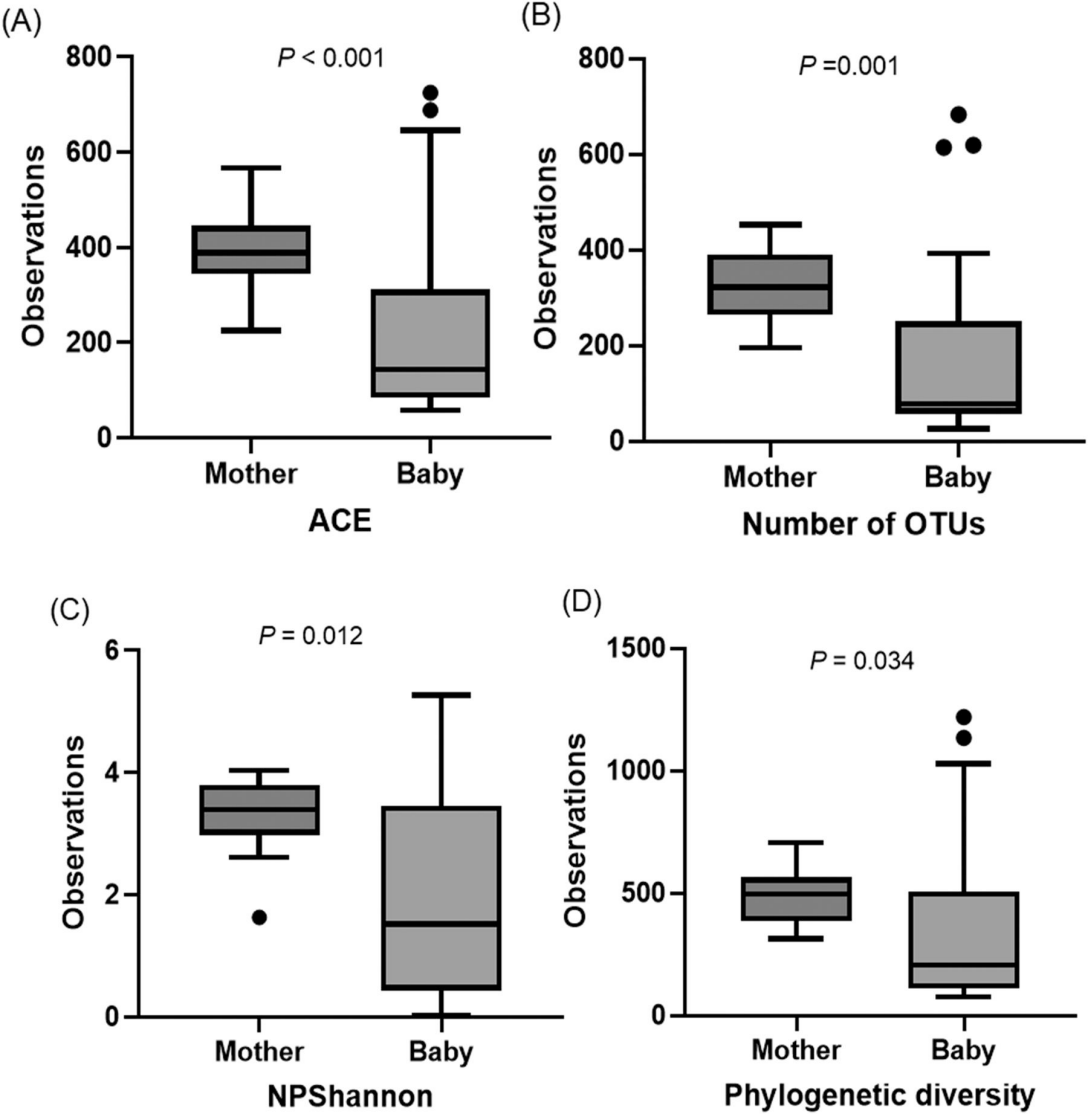
Alpha diversity of the gut microbiota in mothers and neonates. Species richness **(A,B)** and diversity indices **(C,D)** are shown for maternal (Mother, *n* = 21) and neonatal (Baby, *n* = 25) samples. Higher values indicate greater within-sample diversity. Group differences between mothers and neonates were assessed using the Wilcoxon rank-sum test. ACE, abundance-based coverage estimator; OTUs, observed operational taxonomic units.

**Figure 3:**
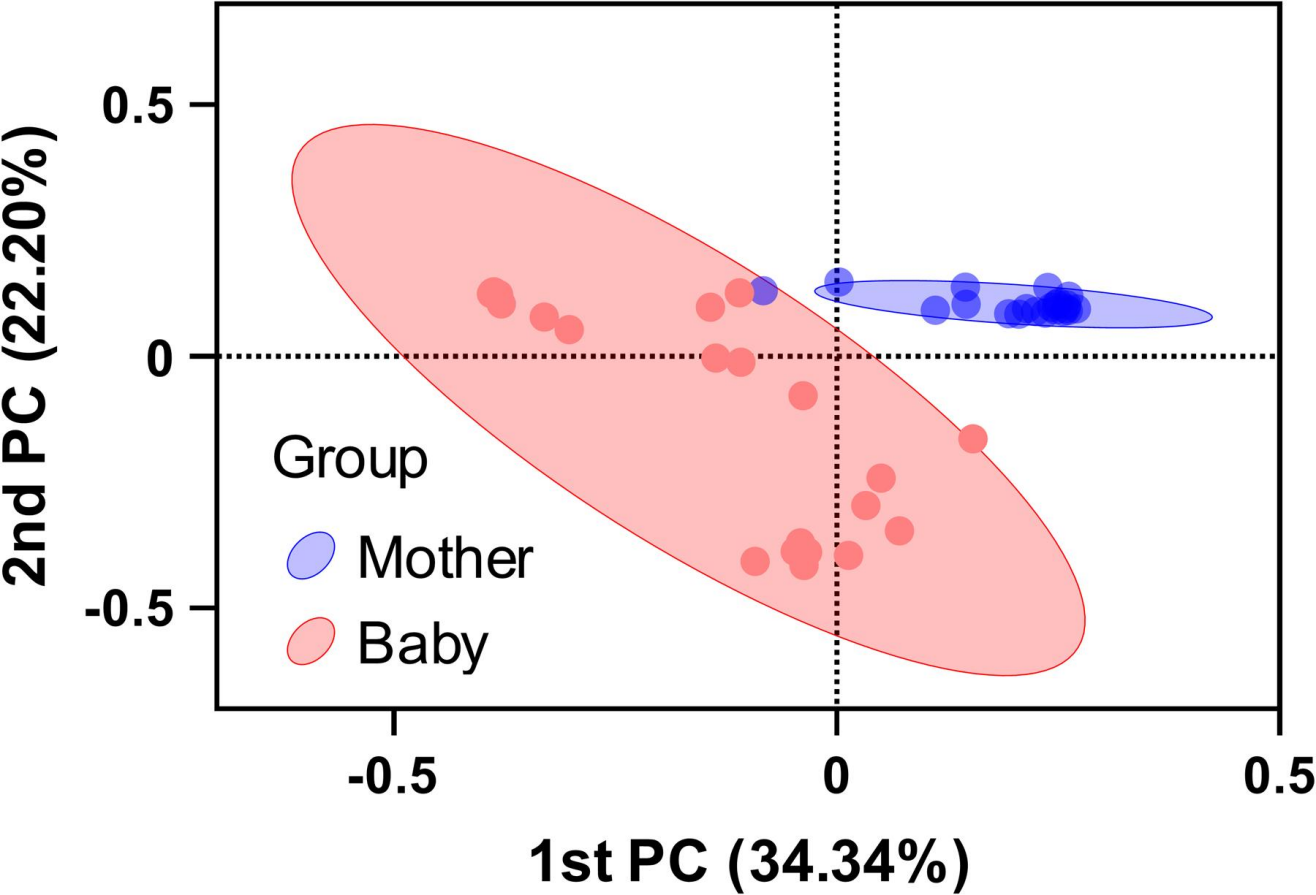
Comparison of gut microbial *β*-diversity at the species-group level. Principal Coordinates Analysis (PCoA) plot based on Jensen-Shannon, combined with K-means clustering, illustrates compositional differences between maternal fecal (blue dots and circle) and early neonatal fecal (pink dots and circle) samples. Each dot represents one individual sample. Overall differences in community composition were statistically significant (PERMANOVA, *P* = 0.001).

**Figure 4:**
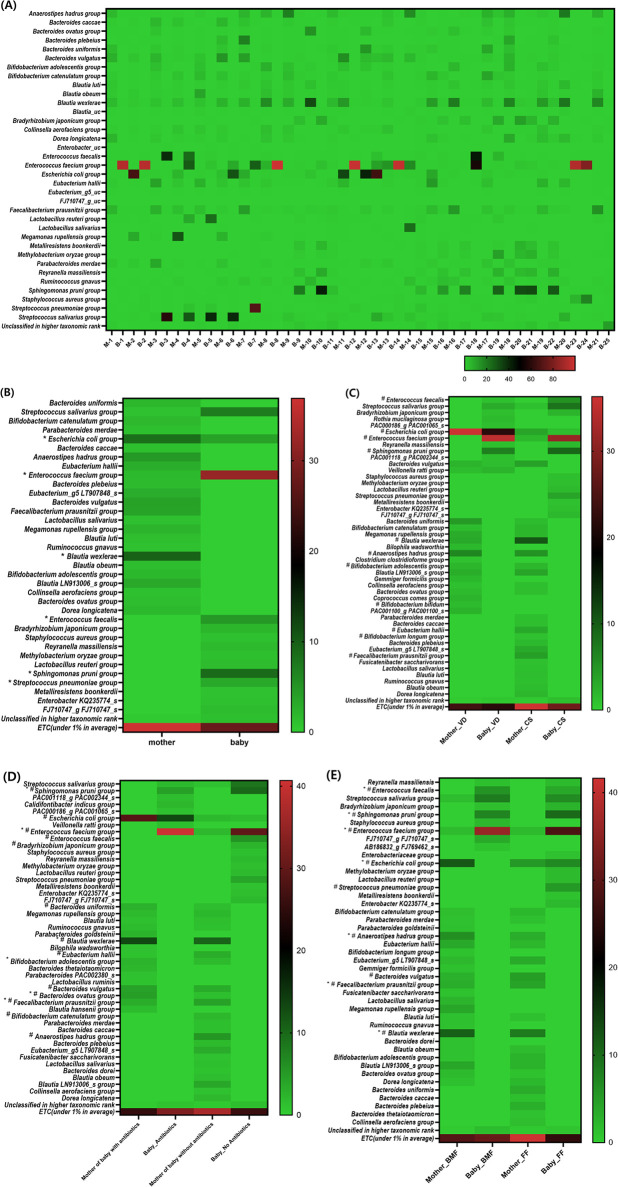
Heatmaps of species-group level in maternal and neonatal early fecal microbiota. **(A)** Individual presentations of maternal and early neonatal fecal microbiota. **(B)** Overall comparisons of maternal and early neonatal fecal microbiota in total. * statistically significantly different species between mother and neonate group **(C)** Comparisons of maternal and early neonatal fecal microbiota according to delivery mode. * statistically different species between mothers and vaginally delivered neonates (VD), # statistically significantly different species between mothers and neonates delivered by Cesarean Section (CS) **(D)** Comparisons of maternal and early neonatal fecal microbiota according to neonatal antibiotics exposure. * statistically different species between mothers and neonates with antibiotics, # statistically significantly different species between mothers and neonates without antibiotics **(E)** Comparisons of maternal and early neonatal fecal microbiota according to feeding mode. *statistically different species between mothers and neonates with breast milk-fed (BMF), # statistically significantly different species between mothers and neonates with formula-fed (FF) Color intensity indicates relative abundance. Subgroup analyses are exploratory because of the limited and unbalanced sample sizes.

We compared *α*-diversity between maternal fecal samples (*n* = 21) and early neonatal fecal samples (*n* = 25) using two complementary metrics,species richness with ACE and the number of OTUs and diversity indices with NPShannon and phylogenetic diversity (Figure. 2). Across both metrics, early neonatal fecal samples exhibited significantly lower *α*-diversity indices than maternal fecal microbiota (*P* < 0.05). PCoA with K-means clustering at the species-group level demonstrated clear separation between maternal fecal and early neonatal fecal microbiota communities in the score plot, and overall differences in community composition were statistically significant by PERMANOVA (*P* = 0.001, Figure 3). To investigate detailed taxonomic concordance at the species-group level between mother and neonates, we generated heat maps from the fecal microbiota of the overall cohort, and clinically relevant subgroups stratified by neonatal antibiotic exposure, delivery mode, and feeding type (Figures 4A–E). In the overall heat maps maternal and early neonatal fecal samples shared some dominant species-group level taxa, but their relative abundance patterns remained distinct. Across the exploratory subgroup heat maps, early neonatal fecal samples repeatedly showed relatively greater representation of several early colonizing taxa, including the *Enterococcus faecium* group and related taxa, whereas maternal fecal samples more often retained taxa enriched in mature adult-associated communities. Similar mother–neonate contrasts were observed across the antibiotic, delivery mode, and feeding subgroups, although the magnitude and composition of these differences varied between strata.

## Discussion

In this study, we examined 21 mother-neonate pairs recruited from a single tertiary medical center to compare maternal fecal microbiota collected shortly before delivery with the offspring's fecal microbiota collected within the first week of life. The primary aim was to determine whether, and to what extent, the maternal gut microbiota influences the initial formation of the neonatal fecal microbiota community. We observed that mothers and neonates shared a broadly similar phylum-level composition dominated by *Firmicutes*. However, early neonatal fecal samples constituted a low-diversity, low-biomass community, exhibiting significantly reduced species richness and lower Shannon indices than maternal stool. Furthermore, maternal fecal and neonatal fecal microbiota communities were clearly segregated indicating the establishment of distinct ecological niches. When mother-neonate pairs were stratified into groups with “concordant” vs. “discordant” dominant phyla and compared across clinical variables, no statistically significant associations were observed with perinatal factors such as pre-pregnancy and gestational weight, gestational diabetes, mode of delivery, or peripartum antibiotic exposure. At species level, early fecal microbiota taxa ratios of neonates delivered vaginally showed no significant differences with mothers compared to the pairs of Cesarean section (CS). However, total ratio of vaginal delivery (VD) was very low in this study. Because several subgroup sizes were limited and unbalanced, species concordances using heat map analyses were interpreted as descriptive exploratory visualizations rather than formal between-subgroup comparisons.

Our findings may reinforce the prevailing concept that the maternal gut microbiota might serves as reservoir for the neonatal intestinal microbiome in some part, while simultaneously demonstrating that the community structure observed in the early neonatal fecal samples collected within the first week of life is not merely a reduced-scale replica of the maternal fecal microbiota. This interpretation is broadly consistent with the strain-level longitudinal analysis by Yassour et al., who demonstrated mother-to-child bacterial transmission during the first months of life and showed that maternal gut-derived strains contribute to early infant colonization, although transmission does not always involve the mother's dominant strain (19). However, unlike the previous study, our analysis was based on 16S rRNA gene profiling of neonatal fecal samples collected within the first week of life, and therefore cannot resolve strain level inheritance or persistence over time. Recent studies using phylogenetic and genomic analyses have shown that maternal gut, vaginal, and skin microbes are transmitted to the infant gut and oral cavity at the strain level, with the maternal gut microbiota contributing most durably over the long term (2, 20). Cohort studies further indicate that maternal gut microbes exhibit statistically significant transmission patterns at the amplicon sequence variant level, with higher transmission rates observed in vaginal deliveries (21, 22). The concordance observed in dominant phyla among certain mother-neonate pairs and no significantly different species in the pairs of VD compared to those of CS in this study may be consistent with this “maternal dependency” hypothesis albeit small number of VD. At the same time, the markedly reduced alpha diversity and the clear separation of maternal and neonatal communities in unweighted distance-based clustering even in the neonatal early fecal samples suggest that, at birth, the early fecal microbiota remains in an early pioneering, or seeding, stage of community assembly (23, 24).

Time-series analyses of the intestinal microbiota indicate that, immediately after birth, facultative anaerobes such as *Escherichia* and *Enterococcus* initially dominate the gut. Over the ensuing months, the microbial community shifts toward a *Bifidobacterium*-centered configuration. Following weaning, *Bacteroides* and members of the phylum *Firmicutes* increase in abundance, and the ecosystem gradually converges toward an adult-like community structure (25, 26). In both the Environmental Determinants of Diabetes in the Young cohort and the Swedish longitudinal study, the gut microbiota develops through three stages—developmental, transitional, and stable—over the first several years of life, achieving adult-like richness and community composition only around five years of age (5, 7). The present study is noteworthy because it examines the earliest point along the neonatal gut microbiota successional pathway—namely, neonatal early fecal samples obtained during the first week of life —and evaluates its relationship with the maternal gut microbiota. The observed low diversity and clear separation of early neonatal microbiota from their mothers indicate that early neonatal fecal samples, although already shaped by environmental, intrauterine milieu, and intrapartum influences, might represents a pre-colonization transmission state. This stage precedes the establishment of a fully developed microbial community. At this early time point, the microbial composition likely reflects a composite signal of DNA from maternally derived bacteria, intrapartum contaminants, and a very small number of pioneer colonizers, and thus has a limited capacity to reliably predict the longer-term developmental trajectory of the gut microbiota over subsequent weeks and months.

The influence of maternal and perinatal factors on initial gut communities has been reported with conflicting results. A recent comprehensive review of maternal microbial inheritance concluded that maternal obesity, dysregulated glucose metabolism, and unhealthy dietary patterns—factors that remodel the maternal gut microbiota—may exert lasting effects on infant gut microbiome composition and function. These effects, in turn, have the potential to program immune, metabolic, and neurodevelopmental axes, ultimately influencing health outcomes in the next generation (27). In a large longitudinal cohort of very preterm infants, maternal pre-pregnancy BMI was identified as the only perinatal factor consistently associated not only with hospital length of stay but also with early childhood gut microbial community structure and functional pathways (28). In contrast, studies focusing on meconium or other low-biomass samples collected immediately after birth have frequently reported weak or inconsistent associations between maternal BMI, mode of delivery, and antibiotic exposure (29–31). In the present study, although the risk ratios of Exp [B] suggested directional trends for maternal BMI, gestational diabetes, and gestational weight gain in relation to concordant dominant phyla between mothers and neonates, none of these associations reached statistical significance. Several factors may account for these findings, including the limited sample size, heterogenous mother-neonate pairs, the relatively coarse categorical outcome defined by concordance of the dominant phylum, and the inherent difficulty of detecting subtle effects in neonatal fecal samples, an extremely low-biomass and contamination-prone specimen. Notably, concordance with the maternal dominant phylum was inconsistent in twin deliveries, suggesting that immediate postnatal environmental exposures and stochastic processes may contribute to the observed variability.

Our exploratory species-group level heat maps provide additional taxonomic granularity beyond the dominant-phylum concordance analysis. Although dominant-phylum concordance was not significantly associated with the examined clinical factors, subgroup heat maps suggested recurrent mother–neonate differences in species-group level abundance patterns within several clinically relevant strata. With respect to delivery mode, species-group level differences between maternal and neonatal samples were less apparent in the vaginal-delivery stratum, but were observed across multiple taxa in the cesarean-delivery stratum. The pattern directionally consistent with prior reports that cesarean delivery is associated with delayed acquisition of beneficial taxa such as *Bifidobacterium* and relative enrichment of taxa such as *Enterococcus* and *Klebsiella* (16, 32). Multiple species-group level differences in proportions between maternal and neonatal samples were observed in both subgroups stratified by neonatal antibiotic exposure. However, less species strains were detected in neonatal fecal samples with antibiotics compared to those without antibiotics. In the feeding-stratified analyses, mother–neonate differences remained apparent in both breast milk-fed and formula-fed, suggesting that the overall maternal–neonatal taxonomic distinction persisted regardless of feeding subgroup in this dataset rather than indicating the absence of a feeding effect. However, these subgroup analyses were exploratory and should not be interpreted as formal between-subgroup comparisons because of the limited and uneven subgroup sizes.

Among these perinatal factors, mode of delivery and antibiotic exposure remain among the best-established determinants of gut microbiota trajectories from birth through infancy. Large birth cohorts have shown that infants delivered via CS experience delayed colonization by *Bacteroides* strains compared with vaginally delivered infants. During the neonatal period, CS-delivered infants' intestinal communities are dominated by opportunistic, hospital-associated taxa such as *Enterococcus*, *Enterobacter*, and *Klebsiella*, whose relative abundance remains significantly higher than that in vaginally delivered infants throughout infancy (33). Previous studies have linked the composition of maternal vaginal and gut microbiomes to fetal and neonatal gut communities. However, most of these studies examined stool samples collected after the first postnatal week, and datasets directly comparing maternal stool with first-week neonatal fecal samples, as in the present study, remain relatively scarce. Given the predominance of CS delivery and the limited number of vaginal births in our cohort, the absence of clear differences according to dominant phylum or several species ratio concordance among the pairs should be interpreted cautiously and may reflect limited statistical power, as well as the fact that the analyzed early neonatal fecal samples were collected within the first week of life, when feeding and environmental exposures may already have influenced microbial composition.

Several limitations must be acknowledged in this study. The study was limited by a small sample size, with clinically heterogenous participants recruited from a single center and representing a relatively homogeneous ethnic background, which constrains the generalizability of the findings. High-risk exposures such as maternal obesity and major obstetric complications were relatively uncommon, reducing the statistical power to detect their potential effects. CS was the predominant mode of delivery in this study (85.7%, *n* = 18), exceeding the national average of approximately 58% reported for Korea in 2023 (34). The high proportion of CS delivery may have limited our ability to fully assess the contribution of vaginal birth to mother-infant microbial transmission, a limitation similarly reported in other studies requiring practical access to maternal feces at delivery (35). Although this was a pragmatic tertiary-center cohort and included neonates born at late-term or term gestation were heterogenous, we could obtain fecal samples from less vulnerable neonatal population compared to early preterm or very low birth weight infants. In addition, we performed only 16S rRNA gene-based relative abundance profiling, which precluded assessment of strain-level transmission and functional pathway dynamics. Finally, because early neonatal fecal samples remain susceptible to environmental contamination, residual contamination cannot be entirely excluded for certain low-abundance taxa, despite rigorous preprocessing and inclusion of negative controls.

Despite these limitations, our study several strengths. The study was performed prospectively and directly examines the relationship between maternal gut microbiota and early neonatal fecal samples at the dyadic mother-infant level. Based our results, the intuitive assumption—that maternal gut microbiota overwhelmingly dictates early fecal community structure—may be only partially valid. Early neonatal fecal samples collected within the first week of life exhibited a broadly similar phylum-level profile to maternal stool; however, in terms of alpha diversity and taxonomic composition, it represents a distinct, low-complexity community whose configuration cannot be fully explained by maternal clinical variables alone. By characterizing the microbiota of mother-neonate pairs, including maternal stool at delivery and neonatal fecal samples, this study contributes to understanding ongoing controversies surrounding the initial assembly of the neonatal gut microbial community. Future studies in larger cohorts should integrate multi-site maternal microbiome (gut, vagina, oral cavity, and skin) with meconium and subsequent infant stools to resolve strain-level transmission and functional potential. Such investigations should also evaluate how modifiable maternal factors—including dietary patterns, weight management, antibiotic exposure, and infant feeding practices—shape gut microbiota development and downstream health outcomes throughout infancy and early childhood. Ultimately, stronger evidence may enable microbiota-informed strategies along the maternal-fetal-neonatal axis to guide immune, metabolic, and neurodevelopmental trajectories of the next generation toward a healthier course.

## Conclusions

In this single-center prospective cohort of 21 mother–newborn pairs, early neonatal fecal samples collected within the first week of life showed markedly reduced richness and diversity compared with maternal fecal microbiota and formed distinct community clusters. Although Firmicutes predominated in both mothers and neonates, only 57% of pairs shared the same dominant phylum, and concordance of phyla or species among the pairs was not explained by delivery mode, feeding type, maternal BMI, gestational diabetes, or postnatal antibiotic exposure. These findings indicate that the maternal gut represents a partial reservoir for early neonatal intestinal seeding, but first-week neonatal fecal samples capture an early, low-biomass community shaped by additional stochastic and perinatal influences. Larger, multi-site, multi-omics studies with strain-level resolution and rigorous contamination control are needed to define transmission routes and identify modifiable maternal factors that support healthy microbiota maturation.

## Data Availability

The original contributions presented in the study are publicly available. This data can be found here: The data presented in the study are deposited in the figshare.com repository, available here: https://figshare.com/s/042c92643b9d5cedcbb0.

## References

[1] Delaroque C Chassaing B. Microbiome in heritage: how maternal microbiome transmission impacts next generation health. Microbiome. (2025) 13(1):1–17. 10.1186/s40168-025-02186-8 41013822 PMC12465890

[2] Ferretti P Pasolli E Tett A Asnicar F Gorfer V Fedi S. Mother-to-Infant microbial transmission from different body sites shapes the developing infant gut microbiome. Cell Host Microbe. (2018) 24(1):133–45. e5. 10.1016/j.chom.2018.06.005 30001516 PMC6716579

[3] Belkaid Y Hand TW. Role of the microbiota in immunity and inflammation. Cell. (2014) 157(1):121–41. 10.1016/j.cell.2014.03.011 24679531 PMC4056765

[4] Rowland I Gibson G Heinken A Scott K Swann J Thiele I. Gut microbiota functions: metabolism of nutrients and other food components. Eur J Nutr. (2018) 57(1):1–24. 10.1007/s00394-017-1445-8 28393285 PMC5847071

[5] Roswall J Olsson LM Kovatcheva-Datchary P Nilsson S Tremaroli V Simon MC. Developmental trajectory of the healthy human gut microbiota during the first 5 years of life. Cell Host Microbe. (2021) 29(5):765–76. e3. 10.1016/j.chom.2021.02.021 33794185

[6] Koenig JE Spor A Scalfone N Fricker AD Stombaugh J Knight R. Succession of microbial consortia in the developing infant gut microbiome. Proc Natl Acad Sci USA. (2011) 108(supplement_1):4578–85. 10.1073/pnas.1000081107 20668239 PMC3063592

[7] Stewart CJ Ajami NJ O’Brien JL Hutchinson DS Smith DP Wong MC. Temporal development of the gut microbiome in early childhood from the teddy study. Nature. (2018) 562(7728):583–8. 10.1038/s41586-018-0617-x 30356187 PMC6415775

[8] Yatsunenko T Rey FE Manary MJ Trehan I Dominguez-Bello MG Contreras M. Human gut microbiome viewed across age and geography. nature. (2012) 486(7402):222–7. 10.1038/nature11053 22699611 PMC3376388

[9] Fan Y Pedersen O. Gut microbiota in human metabolic health and disease. Nat Rev Microbiol. (2021) 19(1):55–71. 10.1038/s41579-020-0433-9 32887946

[10] Tan J McKenzie C Potamitis M Thorburn AN Mackay CR Macia L. The role of short-chain fatty acids in health and disease. Adv Immunol. (2014) 121:91–119. 10.1016/B978-0-12-800100-4.00003-9 24388214

[11] Pammi M Cope J Tarr PI Warner BB Morrow AL Mai V. Intestinal dysbiosis in preterm infants preceding necrotizing enterocolitis: a systematic review and meta-analysis. Microbiome. (2017) 5(1):31. 10.1186/s40168-017-0248-8 28274256 PMC5343300

[12] Cryan JF O'Riordan KJ Cowan CS Sandhu KV Bastiaanssen TF Boehme M. The Microbiota-gut-brain axis. Physiol Rev. (2019) 99(4):1877–2013. 10.1152/physrev.00018.2018 31460832

[13] Cryan JF. Microbiome and brain development: a tale of two systems. Ann Nutr Metab. (2025) 81:34–45. 10.1159/000544950 40064155

[14] Delaroque C Bonazzi E Huillet M Ellero-Simatos S Hao F Patterson A. Maternal diet alters offspring’s early life host-microbiota communication through goblet cells, resulting in long-lasting diseases susceptibility. *bioRxiv*:2024.07. 05.602179 (2024). 10.1038/s41467-025-62397-3 PMC12307616 40730751

[15] Pabst O Slack E. Iga and the intestinal microbiota: the importance of being specific. Mucosal Immunol. (2020) 13(1):12–21. 10.1038/s41385-019-0227-4 31740744 PMC6914667

[16] Reyman M van Houten MA van Baarle D Bosch AA Man WH Chu MLJ. Impact of delivery mode-associated gut microbiota dynamics on health in the first year of life. Nat Commun. (2019) 10(1):4997. 10.1038/s41467-019-13014-7 31676793 PMC6825150

[17] Dos Santos SJ Pakzad Z Albert AY Elwood CN Grabowska K Links MG. Maternal vaginal microbiome composition does not affect development of the infant gut microbiome in early life. Front Cell Infect Microbiol. (2023) 13:1144254. 10.3389/fcimb.2023.1144254 37065202 PMC10097898

[18] Yoon S-H Ha SM Kwon S Lim J Kim Y Seo H. Introducing ezbiocloud: a taxonomically united database of 16s rrna gene sequences and whole-genome assemblies. Int J Syst Evol Microbiol. (2017) 67(5):1613–7. 10.1099/ijsem.0.001755 28005526 PMC5563544

[19] Yassour M Jason E Hogstrom LJ Arthur TD Tripathi S Siljander H. Strain-level analysis of mother-to-child bacterial transmission during the first few months of life. Cell Host Microbe. (2018) 24(1):146–54.e4. 10.1016/j.chom.2018.06.007 30001517 PMC6091882

[20] Xie H Meng L Duan X Liang X Huang T Ma G. Establishment of the early gut microbiota in vaginally delivered infants: the influence of maternal gut microbiota outweighs vaginal microbiota. Microbiol Spectr. (2025) 13(9):e01775–-25. 10.1128/spectrum.01775-25 40792492 PMC12403616

[21] Caprara GL von Ameln Lovison O Martins AF Bernardi JR Goldani MZ. Gut microbiota transfer evidence from mother to newborn. Eur J Pediatr. (2024) 183(2):749–57. 10.1007/s00431-023-05341-1 37987847

[22] Iqbal F Shenoy PA Lewis LES Siva N Purkayastha J Eshwara VK. Influence of perinatal antibiotic on neonatal gut microbiota: a prospective cohort study. BMC Pediatr. (2025) 25(1):560. 10.1186/s12887-025-05907-y 40685344 PMC12278547

[23] Hu J Nomura Y Bashir A Fernandez-Hernandez H Itzkowitz S Pei Z. Diversified microbiota of meconium is affected by maternal diabetes Status. PLoS One. (2013) 8(11):e78257. 10.1371/journal.pone.0078257 24223144 PMC3819383

[24] He Q Kwok LY Xi X Zhong Z Ma T Xu H. The meconium microbiota shares more features with the amniotic fluid microbiota than the maternal fecal and vaginal microbiota. Gut Microbes. (2020) 12(1):1794266. 10.1080/19490976.2020.1794266 32744162 PMC7524391

[25] Beller L Deboutte W Falony G Vieira-Silva S Tito RY Valles-Colomer M. Successional stages in infant gut microbiota maturation. MBio. (2021) 12(6):e01857–21. 10.1128/mbio.01857-21 34903050 PMC8686833

[26] Wampach L Heintz-Buschart A Hogan A Muller EE Narayanasamy S Laczny CC. Colonization and succession within the human gut microbiome by archaea, bacteria, and microeukaryotes during the first year of life. Front Microbiol. (2017) 8:738. 10.3389/fmicb.2017.00738 28512451 PMC5411419

[27] Grech A Collins CE Holmes A Lal R Duncanson K Taylor R. Maternal exposures and the infant gut microbiome: a systematic review with meta-analysis. Gut Microbes. (2021) 13(1):1897210. 10.1080/19490976.2021.1897210 33978558 PMC8276657

[28] Toubon G Patin C Delannoy J Rozé JC Barbut F Ancel P-Y. Very preterm gut microbiota development from the first week of life to 3.5 years of age: a prospective longitudinal multicenter study. Microbiol Spectr. (2025) 13(4):e01636–24. 10.1128/spectrum.01636-24 39969235 PMC11960047

[29] Turunen J Tejesvi MV Paalanne N Pokka T Amatya SB Mishra S. Investigating prenatal and perinatal factors on meconium microbiota: a systematic review and cohort study. Pediatr Res. (2024) 95(1):135–45. 10.1038/s41390-023-02783-z 37591927 PMC10798900

[30] Chu DM Antony KM Ma J Prince AL Showalter L Moller M. The early infant gut microbiome varies in association with a maternal high-fat diet. Genome Med. (2016) 8(1):77. 10.1186/s13073-016-0330-z 27503374 PMC4977686

[31] Chang YS Li CW Chen L Wang XA Lee MS Chao YH. Early gut microbiota profile in healthy neonates: microbiome analysis of the first-pass meconium using next-generation sequencing technology. Children. (2023) 10(7):1260. 10.3390/children10071260 37508757 PMC10377966

[32] Catassi G Mateo SG Occhionero AS Esposito C Giorgio V Aloi M. The importance of gut microbiome in the perinatal period. Eur J Pediatr. (2024) 183(12):5085–101. 10.1007/s00431-024-05795-x 39358615 PMC11527957

[33] Shao Y Forster SC Tsaliki E Vervier K Strang A Simpson N. Stunted microbiota and opportunistic pathogen colonization in caesarean-section birth. Nature. (2019) 574(7776):117–21. 10.1038/s41586-019-1560-1 31534227 PMC6894937

[34] Kim S Oh JW Yun JW. Narrative review on the trend of childbirth in South Korea and feasible intervention to reduce cesarean section rate. J Korean Soc Matern Child Health. (2023) 27(1):1–13. 10.21896/jksmch.2023.27.1.1

[35] Cho KH Kwon Y Kasani PH Lee SG Jeong SJ. Influence of maternal weight dynamics prior to and throughout gestation on early infant gut microbiome colonization. Microb Ecol. (2025) 88(1):1–11. 10.1007/s00248-025-02520-5 40261360 PMC12014846

